# Robust α-Fe_2_O_3_@TiO_2_ Core–Shell Structures With Tunable Buffer Chambers for High-Performance Lithium Storage

**DOI:** 10.3389/fchem.2022.866369

**Published:** 2022-04-07

**Authors:** Chunyuan Pian, Weichao Peng, Haoyu Ren, Chao Ma, Yun Su, Ruixia Ti, Xiuyu Chen, Lixia Zhu, Jingjing Liu, Xinzhi Sun, Bin Wang, Bingxuan Niu, Dapeng Wu

**Affiliations:** ^1^ School of Physics and Electronic Engineering, Xinxiang University, Xinxiang, China; ^2^ Key Laboratory for Biomedical Effects of Nanomaterials and Nanosafety, Institute of High Energy Physics, Chinese Academy of Sciences, Beijing, China; ^3^ Collaborative Innovation Centre of Henan Province for Green Manufacturing of Fine Chemicals, Key Laboratory of Green Chemical Media and Reactions, Ministry of Education, School of Chemistry and Chemical Engineering, Henan Normal University, Xinxiang, China; ^4^ School of Mechanical and Electrical Engineering, Xinxiang University, Xinxiang, China; ^5^ Collage of Pharmacy, College of Biomedical Engineering, Xinxiang Medical University, Xinxiang, China; ^6^ School of Environment, Henan Normal University, Xinxiang, China

**Keywords:** α-Fe_2_O_3_@TiO_2_, core-shell structures, tunable buffer chambers, cycle stability, lithium storage

## Abstract

α-Fe_2_O_3_ has high potential energy storage capacity and can serve as a green and low-cost anode material for lithium-ion batteries. However, α-Fe_2_O_3_ suffers large volume expansion and pulverization. Based on DFT calculations, TiO_2_ can effectively maintain the integrity of the crystal structure during the discharge/charge process. Well-defined cubic α-Fe_2_O_3_ is coated with a TiO_2_ layer using the hydrothermal method with the assistance of oxalic acid surface treatment, and then α-Fe_2_O_3_@TiO_2_ with tunable buffer chambers is obtained by altering the hydrochloric acid etching time. With the joint efforts of the buffer chamber and the robust structure of the TiO_2_ layer, α-Fe_2_O_3_@TiO_2_ alleviates the expansion of α-Fe_2_O_3_ during the discharge/charge process. The optimized sample (FT-1h) achieves good cycling performance. The reversible specific capacity remains at 893.7 mA h g^-1^, and the Coulombic efficiency still reaches up to 98.47% after 150 cycles at a current density of 100 mA g^−1^. Furthermore, the reversible specific capacity can return to 555.5 mA h g^−1^ at 100 mA g^−1^ after cycling at a high current density. Hence, the buffer chamber and the robust TiO_2_ layer can effectively improve the cycling stability and rate performance of α-Fe_2_O_3_.

## Introduction

With the rapid development of portable electronic devices and electric vehicles, the demand for batteries with higher energy densities, greater safety, and longer cycling lifetimes has become extremely urgent (Duffner et al., 2021; Wang et al., 2022). Lithium-ion batteries (LIBs) have been widely used over the past few years and have been increasingly studied (Kim et al., 2019; Guo and Li, 2021). The ideal anode material should have a much higher cycle life and a moderate potential plateau (Jin et al., 2021). Transition metal oxides (MO_*x*_, where M represents Fe, Co, Mn, Ni, etc.) are usually used as anode materials in lithium-ion batteries due to their high theoretical specific capacity (Li et al., 2021a; Li Q. et al., 2021). α-Fe_2_O_3_ has become a promising anode material for Li-ion batteries due to its high theoretical capacity (1,007 mAh g^−1^) and low cost (Kong et al., 2016). However, the poor conductivity and violent volume expansion of α-Fe_2_O_3_ during the discharge/charge progress result in its poor rate and cycling performance as an anode material in Li-ion batteries. To overcome these problems, many researchers have expended considerable efforts to test potential solutions, such as various morphologies [porous quasi-clusters (Li Z. et al., 2019), porous nanotubes (Wang Z. et al., 2018), and one-dimensional mesoporous nanowires (Li et al., 2020)] and conductive carbon material coatings [graphene oxide (Zhang et al., 2019), carbon cloth (Narsimulu et al., 2019), N-doped carbon hybrids (Li et al., 2018), and spherical graphite (Yan et al., 2020)]. Although the rate and cycling performance of α-Fe_2_O_3_ have been improved, they have still not reached satisfactory levels.

TiO_2_ is an essential member of the family of transition metal oxides, which are widely used in photocatalysis (Xu et al., 2017; Wang L. et al., 2018; Shi et al., 2018; Wang et al., 2019a; Wang et al., 2019b), solar cells (Wu et al., 2017), and lithium-ion batteries (Huo et al., 2021). As an anode of lithium-ion batteries, TiO_2_ has good cycling stability and a low volume expansion ratio (less than 4%) (Liang et al., 2020). However, the reversible capacity of TiO_2_ as an anode material in lithium-ion batteries is low, and its theoretical capacity is only 335 mA h g^−1^ (Liang et al., 2020). To improve the specific capacity of TiO_2_, a large number of scientific researchers investigated different structures of TiO_2_, such as porous (Nong et al., 2020), hollow nanosphere (Fan et al., 2021), branch-like (Gao et al., 2019), heterogeneous mesoporous hollow nanocage-in-nanocage, and sandwich structures (Yuan et al., 2019). Additionally, many attempts to combine carbon with TiO_2_ have been made (Ni et al., 2019; Yuan et al., 2021). However, the specific capacity of TiO_2_ has still not been significantly improved.

The combination of α-Fe_2_O_3_ and TiO_2_ may form a new kind of Li-ion battery anode material. α-Fe_2_O_3_ grains grafted onto TiO_2_/carbon nanofibers (CNFs) have been successfully fabricated by electrospinning and vapor–solid reaction (VSR), with the resulting material demonstrating greatly enhanced diffusion kinetics and structural stability for use in lithium-ion batteries (Yang et al., 2019). Robust Fe_2_O_3_ nanoplates have been coated with small TiO_2_ nanoplates, forming a slice-on-slice structure with large voids, with the resulting material exhibiting good Li storage properties (Zhao et al., 2018). A bio-inspired nanotubular TiO_2_/Fe_2_O_3_ composite has been fabricated by using a natural cellulose substance (laboratory filter paper) as the structural scaffold, showing good cycling stability and excellent rate capability (Li S. et al., 2019). Notably, a core–shell structure is an effective means to improve cycling performance (Li et al., 2021). Thus, α-Fe_2_O_3_@TiO_2_ composed of a hollow inner core and an outer shell with massive mesopores has been prepared, thus exhibiting outstanding electrochemical properties (Fu et al., 2015).

Different from the aforementioned materials, a tunable buffer chamber is fabricated in this study. Cubic α-Fe_2_O_3_ is used as the core, and a TiO_2_ layer is coated on this core using the hydrothermal method. With an increasing etching time, α-Fe_2_O_3_@TiO_2_ becomes more hollowed out. Thus, a buffer chamber is formed between α-Fe_2_O_3_ and TiO_2_. With the help of this structure, the volume expansion of α-Fe_2_O_3_ during the discharge/charge process is alleviated when applied as the anode material in lithium-ion batteries, which is also proven by theoretical calculations.

## Theoretical and Experimental Method

### Calculation Method

With the density functional theory (DFT) (Hohenberg, 1964; Kohn and Sham, 1965), the process of Li-ion de/insertion in α-Fe_2_O_3_ and TiO_2_ is studied. Ultrasoft pseudopotentials were used to calculate the interaction of the ionic core and valence electrons for Fe 3d^6^4s^2^, Ti 3s^2^3p^6^3d^2^4s^2^, Li 1s^2^2s^1^, and O 2s^2^p^4^. The exchange and correlation terms were described with the generalized gradient approximation (GGA) of Perdew–Burke–Ernzerhof (PBE) (Perdew et al., 1996; Perdew et al., 1998). Monkhorst–Pack k-point meshes were conducted to address the Brillouin-zone integrations (Monkhorst and Pack, 1976). Convergence criteria were set as follows: the maximum force, the maximum stress, and the maximum displacement on the atom were below 0.1 eV nm^−1^, 0.02 GPa, and 5.0 × 10^–5^ nm, respectively.

A periodic structure was adopted in the calculation. The unit cells of α-Fe_2_O_3_ and anatase TiO_2_ (space group of R-3C and 141/AMD) contained 12 iron atoms, 18 oxygen atoms, four titanium atoms, and eight oxygen atoms (Supplementary Figure S1). Moreover, 380 eV was set as the cutoff energy for both. The number of k-points was set as 6 × 6×2 and 7 × 7×3. The calculated lattices agreed well with the theoretical and experimental data (Finger and Hazen, 1980; Arlt et al., 2000; Zhang et al., 2017; Jenness et al., 2018) (Supplementary Table S1).

### Synthesis of α-Fe_2_O_3_

With vigorous stirring in an oil bath at 75°C, 50 ml of 2.0 mol/L FeCl_3_·6H_2_O solution was added to a round-bottom flask containing 50 ml of 5.4 mol/L NaOH. After stirring for 5 min, a red-brown Fe(OH)_3_ colloid formed in the flask, which was then transferred to a high-temperature and high-pressure PTFE reactor. The hydrothermal reaction was conducted at 100°C for 4 h. After cooling to room temperature, the obtained red precipitate was centrifuged, successively rinsed with deionized water and ethanol three times, and then dried overnight to obtain α-Fe_2_O_3_.

The synthesized α-Fe_2_O_3_ was treated with oxalic acid, for which 8 ml of deionized water was added to a beaker containing 0.2 g of α-Fe_2_O_3_, and then 0.1 g of oxalic acid was added. After shaking at room temperature for 6 h, the red precipitate was obtained by centrifugation and then successively rinsed with deionized water and ethanol three times. After drying overnight, oxalic acid-treated α-Fe_2_O_3_ was obtained.

### Synthesis of Core–Shell α-Fe_2_O_3_@TiO_2_

**FIGURE 1:**
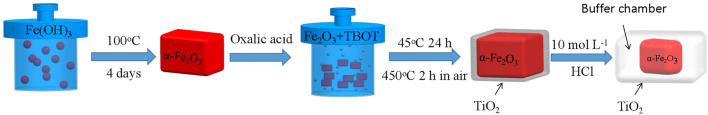
Schematic showing the synthesis of core–shell α-Fe_2_O_3_@TiO_2_.

The core–shell α-Fe_2_O_3_@TiO_2_ synthesis process is illustrated in Figure 1. In a beaker containing 33 ml of absolute ethanol, 0.1 g of oxalic acid-treated α-Fe_2_O_3_ was added, and then, 0.1 ml of concentrated ammonia water was added with stirring and stirred for 5 min. Next, 0.25 ml of tetrabutyl titanate (TBOT) was added with vigorous stirring, and ultrasonication was performed for 40 min. The solution in the beaker was transferred to the PTFE reactor. After hydrothermal reaction at 45°C for 24 h, the solution was cooled to room temperature and then centrifuged. The red precipitate was rinsed with deionized water and ethanol three times and then dried overnight. In an air environment, the product was calcined at 450°C for 2 h to obtain α-Fe_2_O_3_@TiO_2_, and then, 0.1 g α-Fe_2_O_3_@TiO_2_ was added to 25 ml of 10 mol/L HCl and oscillated for 0.5, 1, 2, 4, and 12 h. Then, they were successively rinsed with deionized water and ethanol three times and dried overnight to obtain α-Fe_2_O_3_@TiO_2_ at different etching degrees. The samples were denoted as FT-0.5h, FT-1h, FT-2h, FT-4h, and FT-12h (pure TiO_2_).

### Material Characterization

X-ray diffraction (XRD, Bruker D8, Germany) was used to characterize the synthesized powder samples. The morphology of each sample was observed by field emission scanning electron microscopy (FESEM) and high-resolution transmission electron microscopy (HRTEM, JEOL, JSM-2100, Japan). The component and valence analysis of the synthesized samples were carried out by X-ray photoelectron spectroscopy (XPS, ESCALAB 250, an Al K excitation source, United States). Additionally, HRTEM (TF20, JOEL, 2100F, Japan) was used to map the elemental distribution of the samples. The specific surface area and pore size analyzer (BET, ASiQ Mini QUANTACHROME) was used to determine the porosity of the samples.

### Electrochemical Characterization

The prepared samples were mixed with acetylene black and polyvinylidene fluoride (PVDF) at a weight ratio of 7:2:1 in N-methyl-2 pyrrolidone (NMP). The obtained slurry was uniformly smeared on a 9-μm-thick copper foil and dried in a vacuum oven at 80°C for 10 h, and then a 12-mm-diameter negative plate was cut, and 1 M LiPF_6_, in a mixture of 1:1:1 vol.% ethylene carbonate (EC), diethyl carbonate (DEC), and dimethyl carbonate (DC) was used as the electrolyte (ternary electrolyte LBC305-1, Shenzhen Kejinzida Technology Co., Ltd.). With a 15.6-mm × 0.45-mm lithium sheet as the positive electrode and a Celgard 2325 diaphragm as the battery separator, the prepared negative electrode was assembled into a CR2025 battery in a glove box (Super, Germany Mikrouna) that was filled with argon gas (water concentration <0.1 ppm and oxygen concentration <0.1 ppm). The cycling and rate performance were tested with a Blue Power instrument (Wuhan Blue Electronics Co., Ltd.). An electrochemical workstation (Shanghai Chenhua Instrument Co., Ltd.) was used to obtain cyclic voltammetry (CV) curves, and a different electrochemical workstation (ZAHNER Company, Germany) was used to conduct electrochemical impedance spectroscopy (EIS).

## Results and Discussion

### Calculation Analysis

**FIGURE 2:**
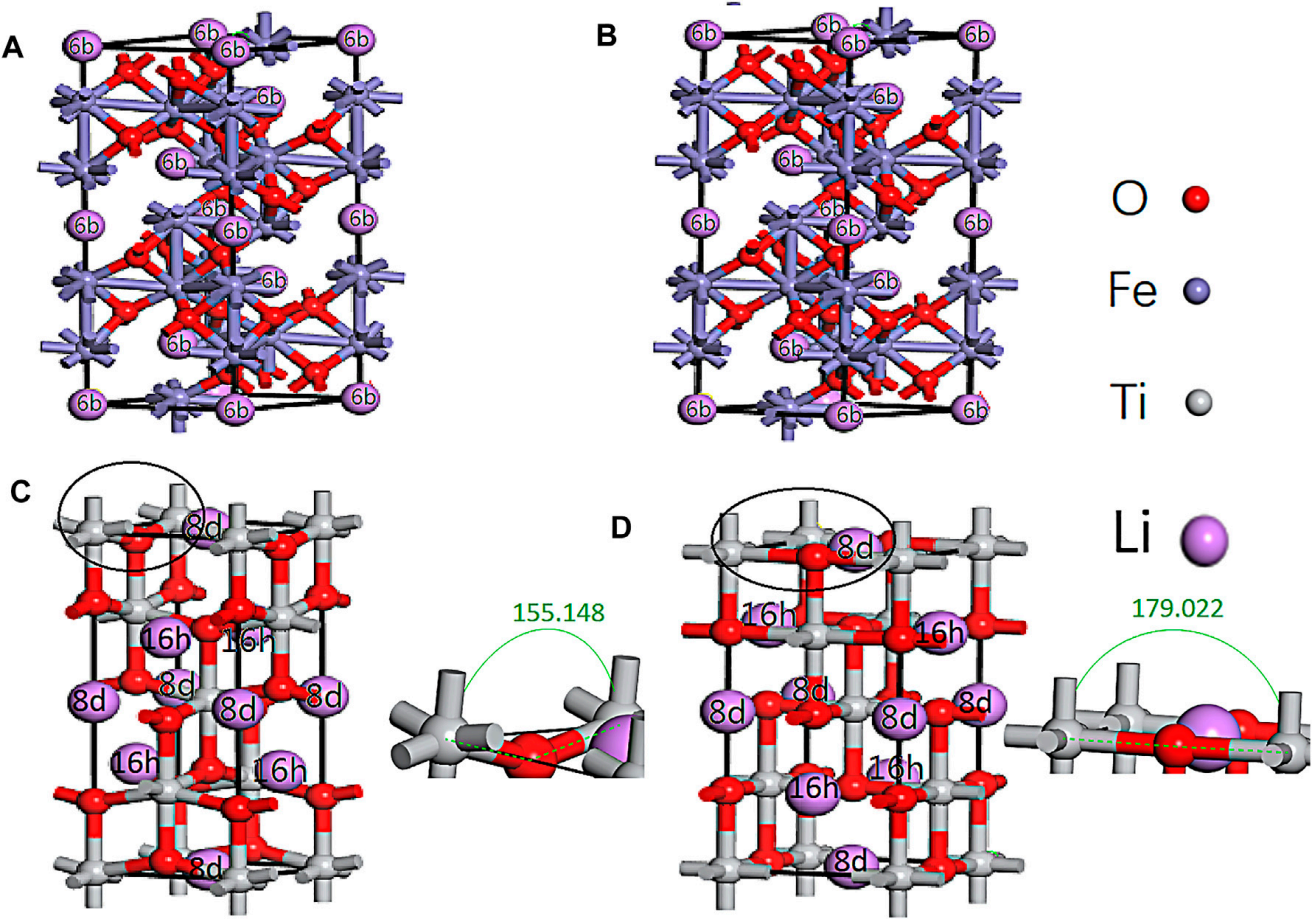
Li inserted in the symmetrical sites of α-Fe_2_O_3_ (6b) and TiO_2_ (16 h, 8 days) before the geometry optimization **(A,B)** and after the geometry optimization **(B,D)**.

According to the symmetry of α-Fe_2_O_3_ and anatase TiO_2_, there are one and two symmetrical positions for Li ions to be inserted into α-Fe_2_O_3_ (6b) and TiO_2_ (16 h, 8 days), respectively (Figure 2). One unit cell of α-Fe_2_O_3_ can accommodate six Li ions, but one unit cell of TiO_2_ can only accommodate four Li ions. Thus, the reaction equation of Li ions and Fe_2_O_3_ is as follows:

$${\text{Fe}}_{2}{\text{O}}_{3}+{\text{Li}}^{+}+{\text{e}}^{-}\to \text{Li}\left({\text{Fe}}_{2}{\text{O}}_{3}\right).$$ (1)

Eq (1) is consistent with Yang et al. (2019). The reaction process of Li ions and TiO_2_ is given in Eq. 2:

$${\text{TiO}}_{2}+{\text{Li}}^{+}+{\text{e}}^{-}↔{\text{LiTiO}}_{2}.$$ (2)

**FIGURE 3:**
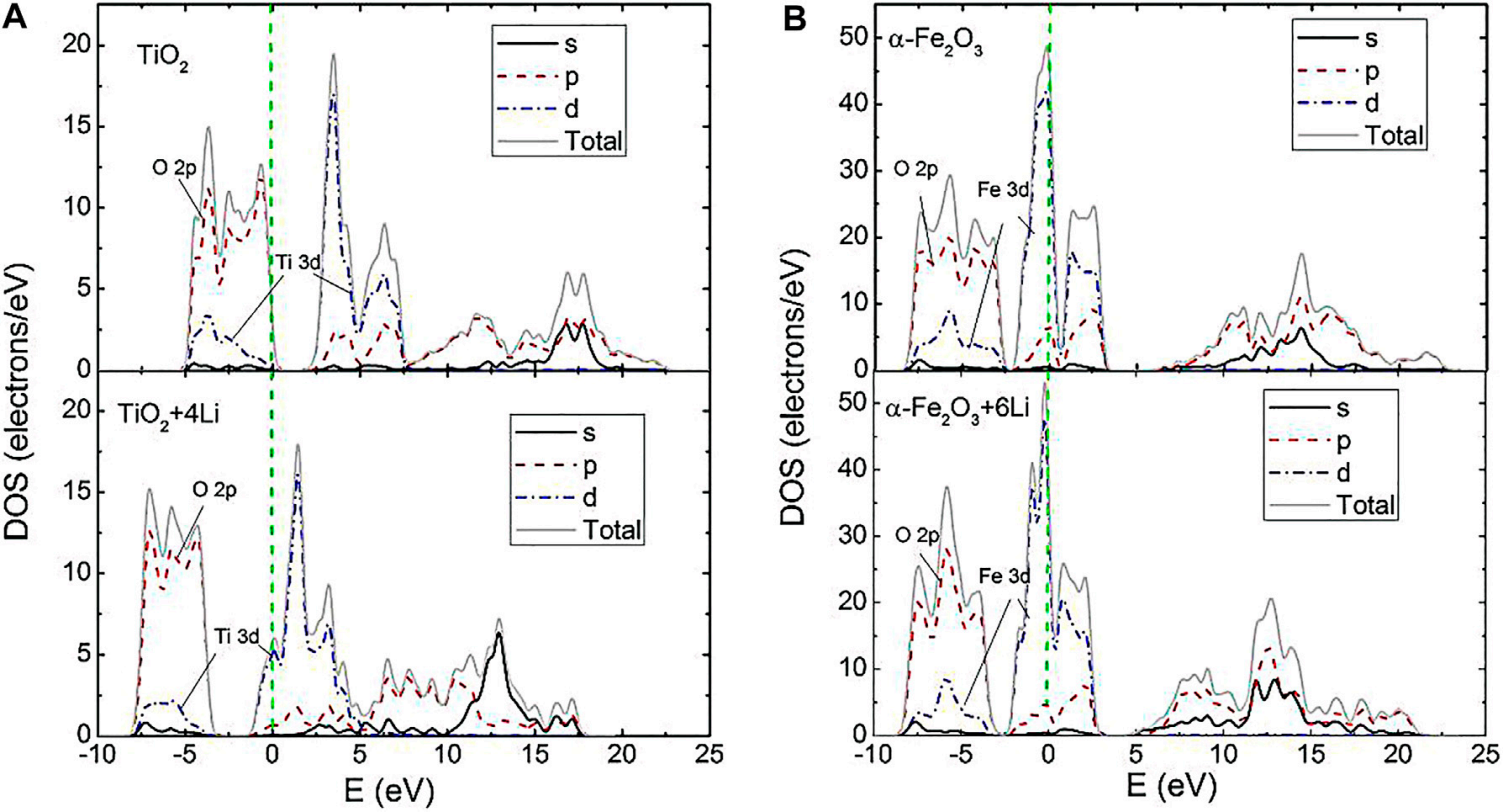
Density of states of TiO_2_ **(A)** and α-Fe_2_O_3_ **(B)** with and without Li inserted at the symmetrical sites. The green dashed line represents a Fermi energy of 0.

From Eq. 2, the theoretical capacity of TiO_2_ can be calculated (335 mA h g^−1^), which is consistent with Liang et al. (2020). After six Li ions were inserted into the α-Fe_2_O_3_ unit cell, the volume expanded by 9.64%. The volume of TiO_2_ expanded by 1.15% after four Li ions were inserted. Moreover, the one, two, three, four, and five and one, two, and three Li ions inserted in the cells at the different sites of α-Fe_2_O_3_ and TiO_2_ were also calculated (Supplementary Table S2). With an increasing number of Li ions inserted in the α-Fe_2_O_3_ cell, the volume continued to expand. However, it was amazing to find that the expansion rate of TiO_2_ always remained below 2.2%. With the same number of Li ions inserted in the cell, the volume expansion of α-Fe_2_O_3_ was larger than that of TiO_2_. There are two reasons for this result: one is that the interstitial vacancy (8 days and 16 h) of TiO_2_ is larger than that of α-Fe_2_O_3_ (6b) and the other is that with Li ions insertion, the angle of Ti-O-Ti (Figures 2C,D) changes from 155.148° to 179.022°, which acts as a buffer. Hence, TiO_2_ can effectively maintain its cell structure. Furthermore, it is believed that with an increasing number of Li ions inserted in α-Fe_2_O_3_ and the combination of O ions, the cell will continue to expand. This mechanism is the reason to coat α-Fe_2_O_3_ with TiO_2_. Moreover, it is not found that the bonds of Ti-O and Fe-O are broken, which can be verified from Figure 2, the population analysis (Supplementary Table S3), and the density of states (DOS) (Figure 3).

The electronic population is usually used to assess the covalent or ionic nature of a bond. A high value of the bond population expresses a strong covalent bond (Aouadi, 2006). Both α-Fe_2_O_3_ and TiO_2_ have two types of bonds, which are marked I and II in Supplementary Figure S1. From Supplementary Table S3, it can be found that the electronic populations of Fe-O I were little larger than those of Fe-O II. Meanwhile, the bond length of Fe-O I was shorter than that of Fe-O II. Nevertheless, the electronic population of Ti-O I was larger than that of Ti-O II, which means Ti-O I had stronger covalent bonds. When Li ions were inserted in α-Fe_2_O_3_ and TiO_2_, the electronic population and bond length increased. Bond length increase can be attributed to volume expansion. Among them, the electronic population of Ti-O I increased the most, which means Ti-O I was enhanced. Instead of breaking, Ti-O I was strengthened.

The DOS of α-Fe_2_O_3_ and TiO_2_ express two main regions near the Fermi level (Figure 3). The more important part is the first region, which is predominantly formed by non-metal O 2p states and Ti 3d (Fe 3d) states located between −10 and 0 eV. This part forms a strong p-d covalent bonding. With the Ti (Fe) and O atoms getting squeezed, it is found that the DOS below the Fermi level move to the low-energy part (the DOS of TiO_2_+4 Li and Fe_2_O_3_+6 Li), which shows that Ti-O and Fe-O are enhanced. Furthermore, the TiO_2_+4 Li DOS move more than the Fe_2_O_3_+6 Li DOS. This result indicates that Ti-O is more enhanced, which agrees well with the electronic population analysis.

### Morphological and Structural Characterization

**FIGURE 4:**
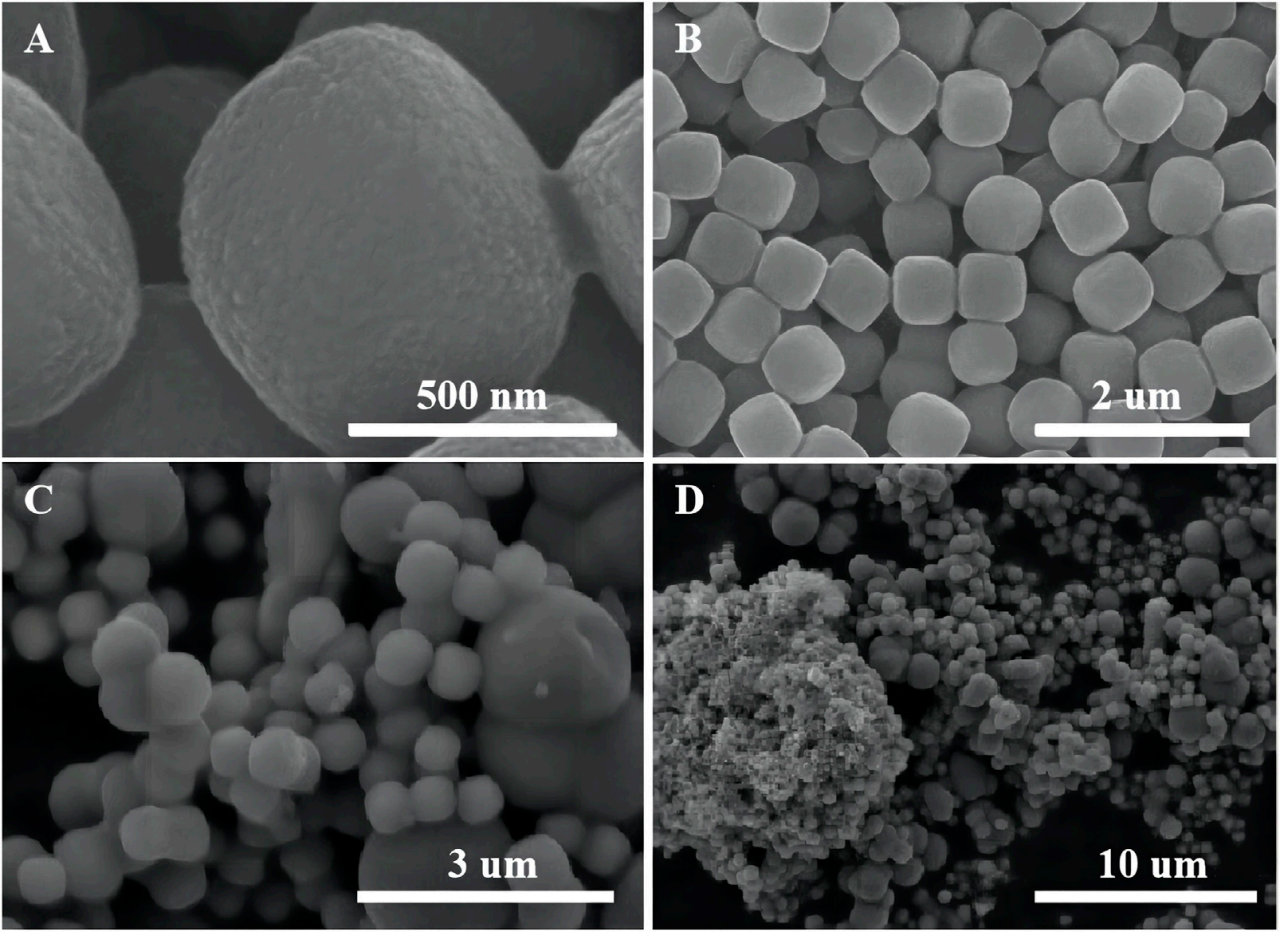
SEM images of α-Fe_2_O_3_ **(A)** and **(B)** and unprocessed α-Fe_2_O_3_ coated with TiO_2_ **(C)** and **(D)**.

The SEM images of α-Fe_2_O_3_ without coating TiO_2_ are shown in Figures 4A,B. α-Fe_2_O_3_ is successfully synthesized during the experiment. The XRD result is consistent with the diffraction peak of α-Fe_2_O_3_ (Haematite, syn) in PDF#33-0664 (Supplementary Figure S2). α-Fe_2_O_3_ has a cubic morphology with relatively smooth surfaces and a size of approximately 400–500 nm. In order to compare the effect of oxalic acid-treated and untreated α-Fe_2_O_3_ coating with TiO_2_, untreated α-Fe_2_O_3_ is used for TiO_2_ layer cladding. According to Figures 4C,D, when untreated α-Fe_2_O_3_ is coated with TiO_2_, it is more prone to heterogeneous nucleation, and agglomeration is easy to occur. After cladding, the homogeneous Fe_2_O_3_ particles become inhomogeneous α-Fe_2_O_3_@TiO_2_ particles.

**FIGURE 5:**
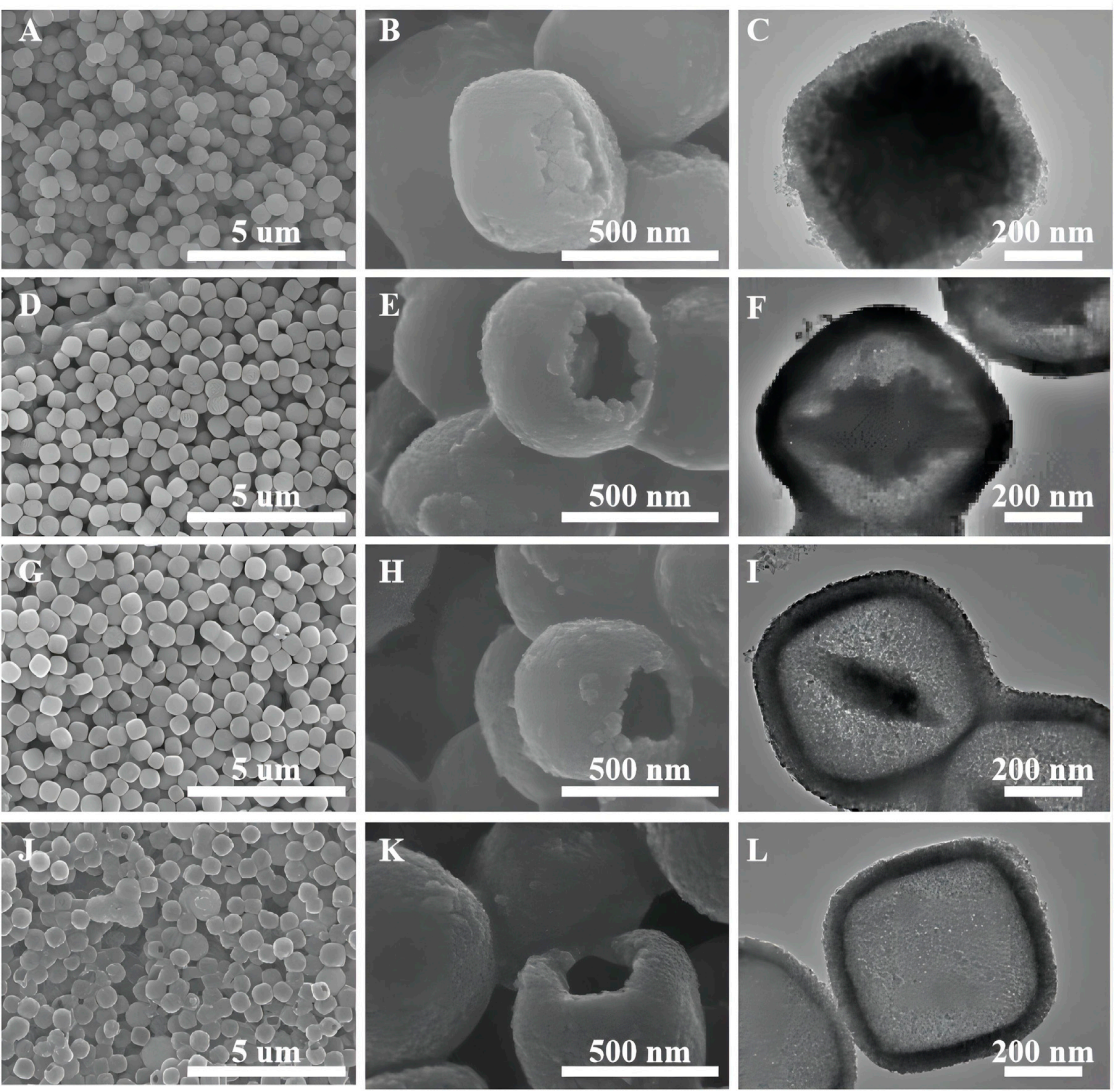
SEM images of oxalic acid-treated α-Fe_2_O_3_ coated with TiO_2_ and etched for different periods of time [FT-0.5h **(A,B)**, FT-2h **(D,E)**, FT-4h **(G,H)**, and FT-12h **(J,K)** (pure TiO_2_)]. TEM images of oxalic acid-treated α-Fe_2_O_3_ coated with TiO_2_ and etched for different periods of time [FT-0.5h **(C)**, FT-2h **(F)**, FT-4h and **(I)**, FT-12h **(L)** (pure TiO_2_)].

Figure 5 shows the samples of oxalic acid-treated α-Fe_2_O_3_ coated with TiO_2_ and etched for different periods of time. Clearly, with an increasing etching time, α-Fe_2_O_3_@TiO_2_ gradually changes from a core–shell structure to a completely hollowed out TiO_2_ structure. The average core–shell sizes of different samples are listed in Supplementary Table S4. With hydrochloric acid etching, the thickness of shell changes a little, reducing from 58 to 45 nm, and maintains at 53 nm during the etching time of 1–4 h, while the core size changes greatly. The vertical diagonal (diagonal 1) changes from 560 to 100 nm. The horizontal diagonal (diagonal 2) changes from 568 to 419 nm. Thus, hydrochloric acid etching mainly aims to Fe_2_O_3_.

**FIGURE 6:**
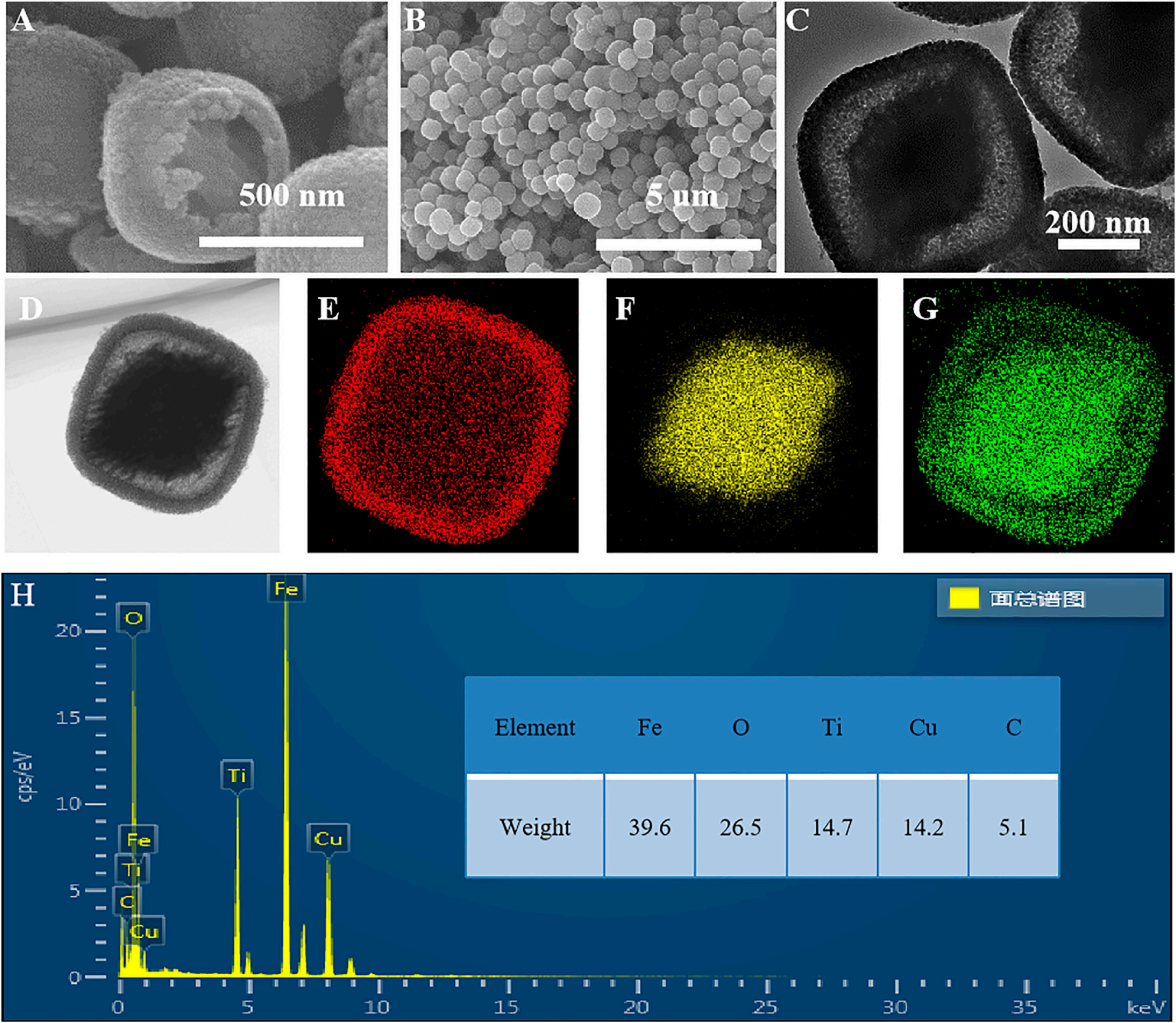
SEM **(A,B)** and TEM-EDS of a single FT-1h α-Fe_2_O_3_@TiO_2_ core–shell structure **(C–H)**.

**FIGURE 7:**
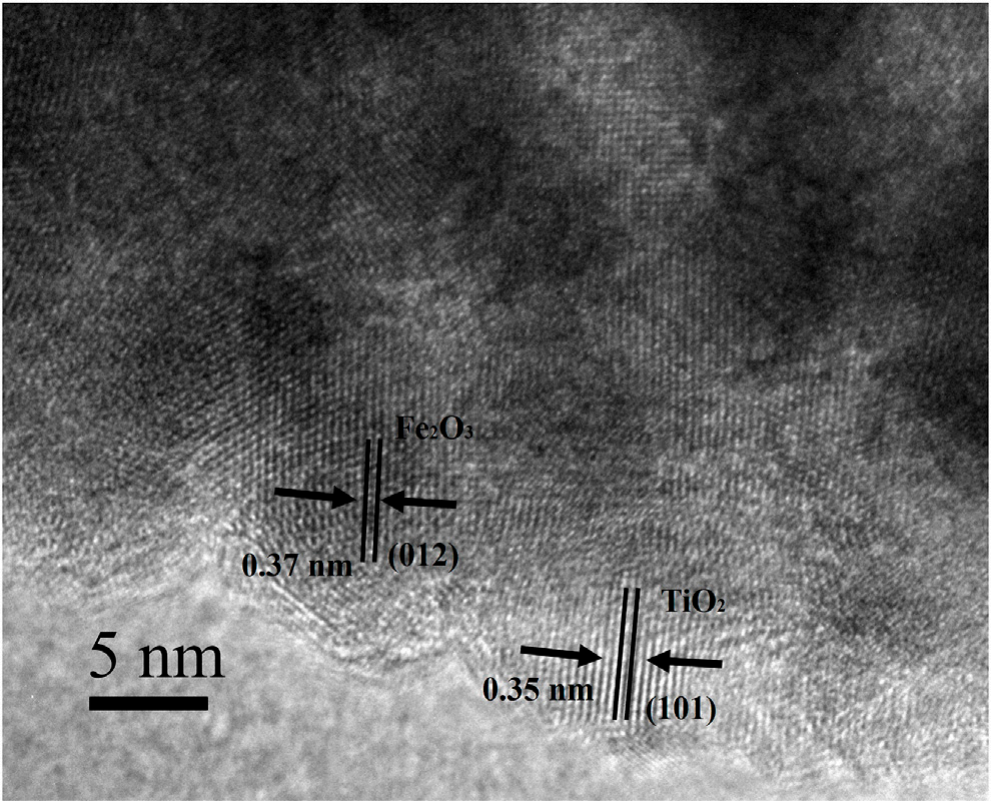
HRTEM image of FT-1h α-Fe_2_O_3_@TiO_2_.

As shown in Figure 6, the sample etched for 1 h (FT-1h) possesses a well-defined core–shell structure with a buffer chamber between the α-Fe_2_O_3_ core and the TiO_2_ shell. From Figure 6A, it can be clearly observed that TiO_2_ is successfully coated on α-Fe_2_O_3_. Furthermore, the TiO_2_ coating layers are very uniform with few ruptures (Figure 6B). In addition, Figure 6C shows that there is a chamber between the α-Fe_2_O_3_ core and the TiO_2_ shell whose thickness is about 80 nm. According to the EDS mapping of a single α-Fe_2_O_3_@TiO_2_ core–shell structure (Figure 6D), Fe, Ti, and O are evenly distributed. The red section in Figure 6E shows that the TiO_2_ layer covers the surface of α-Fe_2_O_3_@TiO_2_, and the yellow part in Figure 6F represents the α-Fe_2_O_3_ core structure. The green section illustrated in Figure 6G refers to O that is jointly contained by α-Fe_2_O_3_ and the TiO_2_ covering layer in a single α-Fe_2_O_3_@TiO_2_ core–shell structure. The composition of composite is further demonstrated by energy-dispersive spectroscopy (EDS), shown in Figure 6H. From the illustration, it shows that the compound is mainly composed of Fe_2_O_3_ and TiO_2_, and Fe_2_O_3_ is the main part. Figure 7 displays the HRTEM image of FT-1h α-Fe_2_O_3_@TiO_2_. The stripes and grids on dark and light are different, which demonstrated that they were assembled by small crystals with measured lattice d-spacings of 0.35 and 0.37 nm, corresponding to the (101) plane of anatase TiO_2_ and the (012) plane of Fe_2_O_3_, respectively. So, it can be proven that TiO_2_ is uniformly dispersed on the surface of Fe_2_O_3_.

**FIGURE 8:**
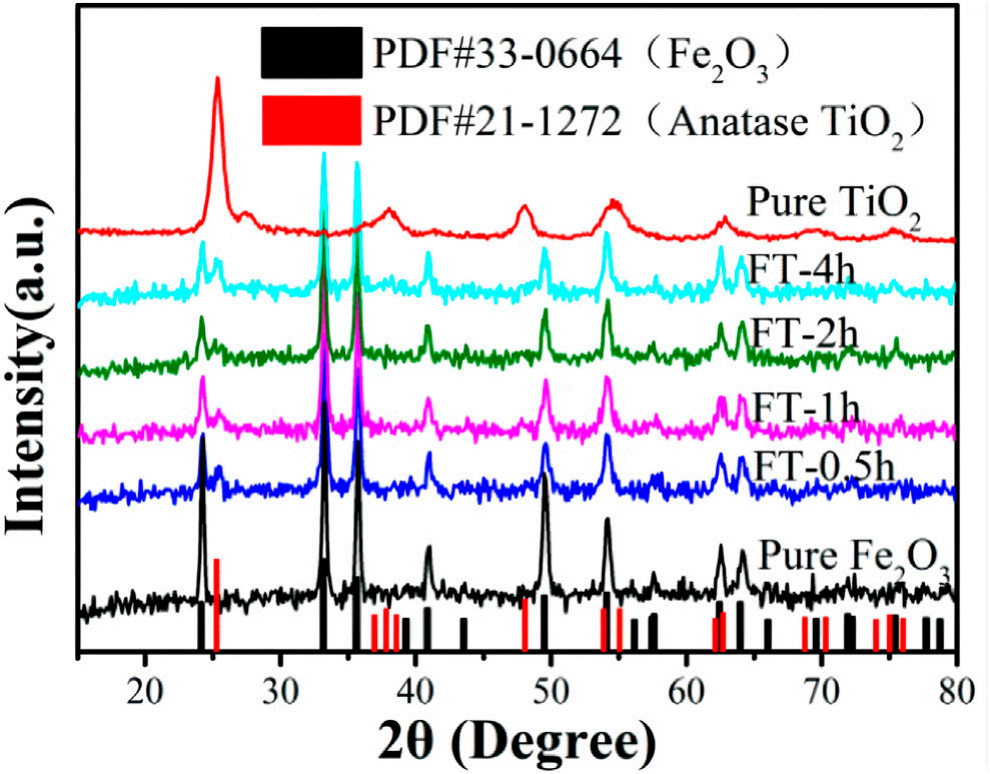
XRD patterns of pure Fe_2_O_3_, FT-0.5h, FT-1h, FT-2h, FT-4h, and FT-12h (pure TiO_2_).

**FIGURE 9:**
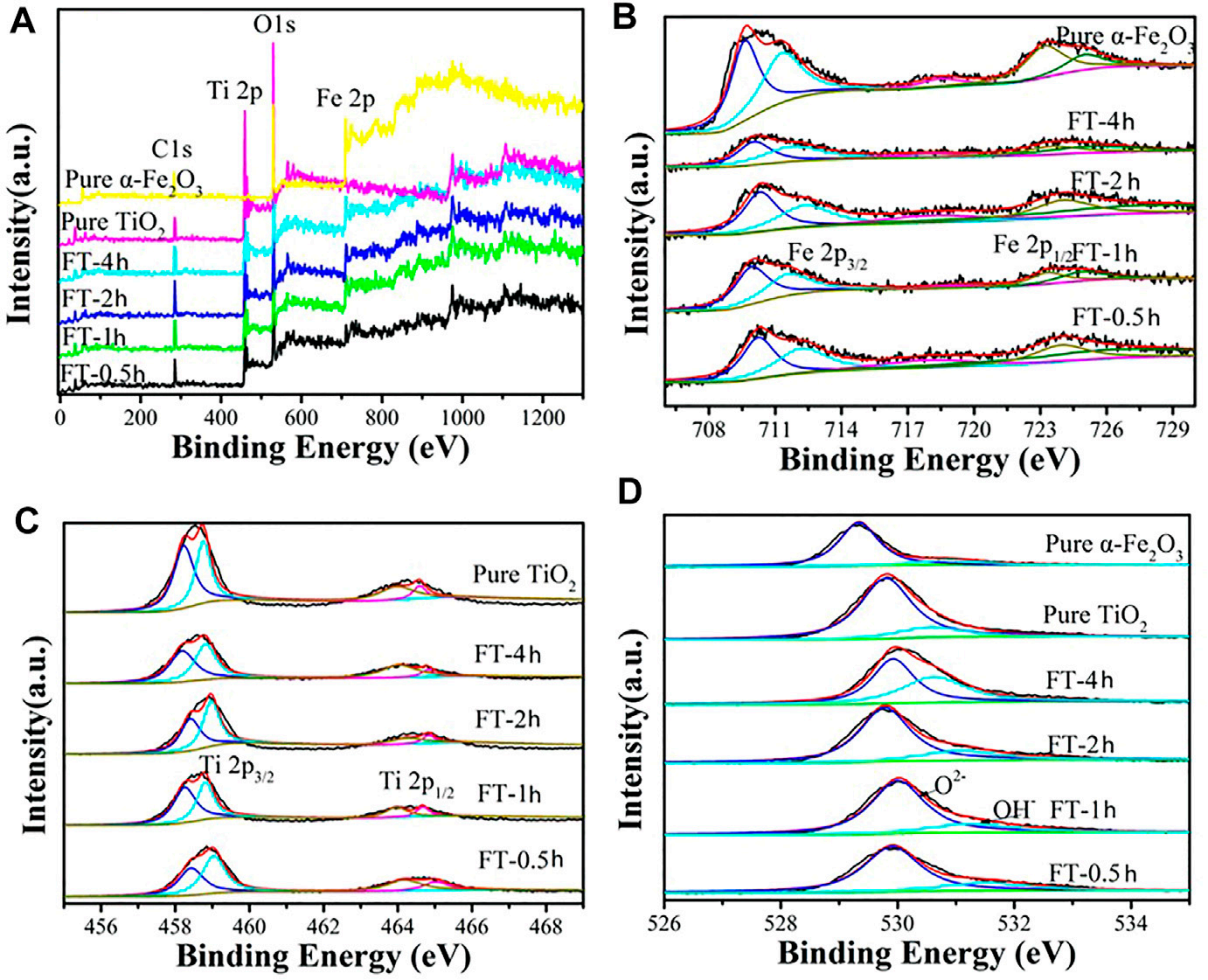
XPS spectra of oxalic acid-treated α-Fe_2_O_3_ coated with TiO_2_ and etched for different periods of time [FT-0.5h, FT-1h, FT-2h, FT-4h, and FT-12h (pure TiO_2_)]: **(A)** survey, **(B)** Fe2p, **(C)** Ti2p, and **(D)** O1s.

The chemical composition and crystal phase of the samples were investigated by XRD analysis. Figure 8 shows that the diffraction peak 2θ = 25.28°, 37.8°, 48.05°, 55.06°, and 62.69° corresponds to the (101), (004), (200), (211), and (204) planes of TiO_2_, which is consistent with PDF#21-1272 (the diffraction peak of anatase). Samples FT-0.5h, FT-1h, FT-2h, and FT-4h present the characteristic peaks of Fe_2_O_3_ and TiO_2_, which expressed that TiO_2_ coating is successfully combined with α-Fe_2_O_3_. Furthermore, it can be observed that the intensity of the characteristic peak at 24.1° of α-Fe_2_O_3_ decreases, and the characteristic peak at 25.3° of TiO_2_ increases with an increasing etching time. In addition, no additional peaks were observed, suggesting high purity of the samples, which shows good agreement with the results of XPS spectra (Figure 9).

The XPS characterization was conducted to examine the chemical composition of samples (FT-0.5h, FT-1h, FT-2h, FT-4h, and FT-12h (pure TiO_2_)). The complete survey spectrum (Figure 9A) of α-Fe_2_O_3_@TiO_2_ samples (FT-0.5h, FT-1h, FT-2h, and FT-4h) reveal the existence of Ti, Fe, and O elements. For the C 1s XPS spectrum, the peak at 285.00 eV is attributed to adventitious carbon (Zhong et al., 2017; Yang et al., 2019). The high-resolution XPS spectrum of Fe 2p was composed of two distinct peaks at 710.90 and 724.40 eV (Figure 9B), which correspond to Fe 2p^2/3^ and 2p^1/2^ with satellite lines. The spectrum is consistent with the characteristic of Fe^3+^ α-Fe_2_O_3_ (Zhong et al., 2017; Yang et al., 2019). In the spectrum of Ti 2p (Figure 9C), the Ti 2p^3/2^ and Ti 2p^1/2^ peaks were located at binding energies of 458.70 and 464.65 eV, respectively, which is in agreement with the value of Ti^4+^ in the TiO_2_ lattice (Zhong et al., 2017; Zhao et al., 2018). The spectrum of the O1s core level is shown in Figure 9D, where binding energy peaks at 531.80 eV originate from bonded hydroxyl groups (Yang et al., 2019). For the broad peak centered at 530.00 eV, it is attributed to metal-bonding in both oxides (Zhao et al., 2018; Yang et al., 2019).

**FIGURE 10:**
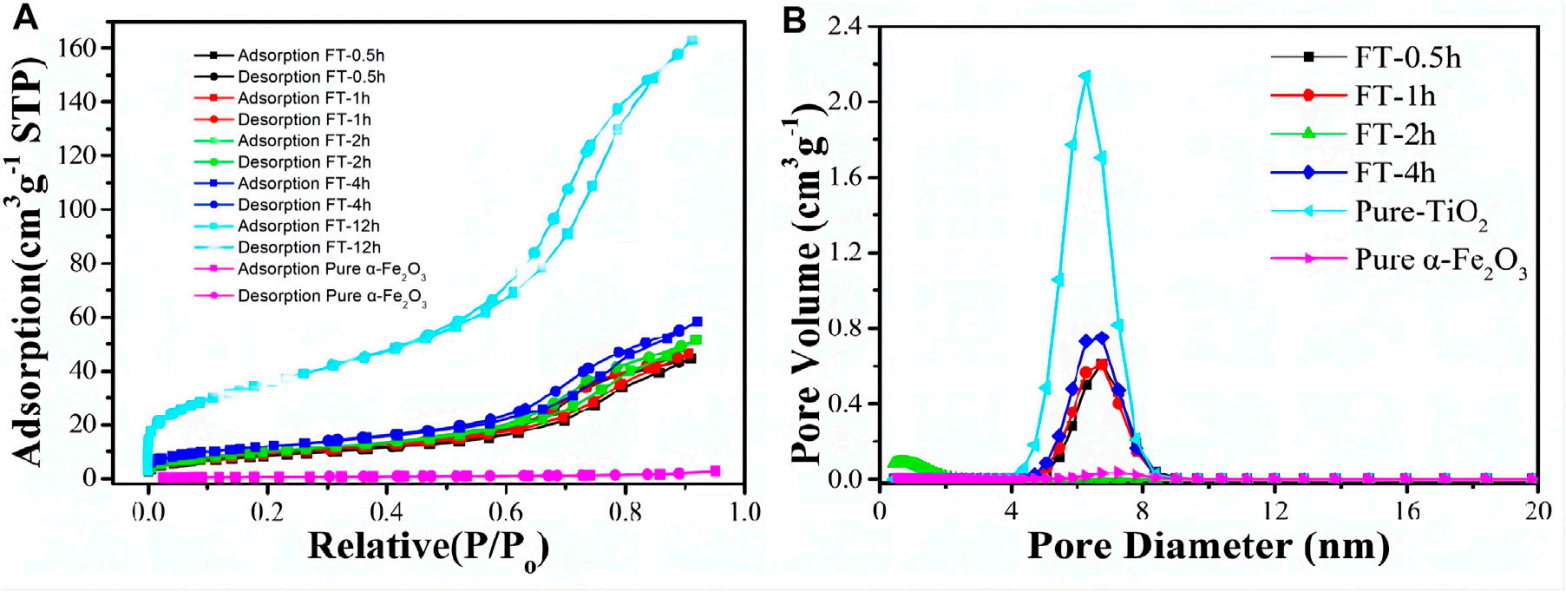
BET figures **(A)** and pore size distribution **(B)** of oxalic acid-treated α-Fe_2_O_3_ coated with TiO_2_ and etched for different periods of time [pure α-Fe_2_O_3_, FT-0.5h, FT-1h, FT-2h, FT-4h, and FT-12h (pure TiO_2_)].

The texture characteristic of α-Fe_2_O_3_@TiO_2_ was further confirmed by N_2_ adsorption/desorption isotherm, as shown in Figure 10. It can be observed from Figure 10A that the specific surface area of pure α-Fe_2_O_3_ was very small, only 2.07 m^3^ g^−1^. After coating with TiO_2_, the specific surface area increased to approximately 30 m^3^ g^−1^, and that is because pure α-Fe_2_O_3_ is smoother and denser than TiO_2_, which can be seen from Figure 4. The surface bulge of TiO_2_ was more obvious than that of pure α-Fe_2_O_3_. After etching for 0.5, 1, 2, 4, and 12 h (pure TiO_2_), more pores formed. The specific surface area increased to 32.1, 34.39, 36.96, 43.845, and 131.91 m^3^ g^−1^. The isotherms were identified as IUPAC type IV, which is characteristic of mesoporous materials. The pore size distribution obtained from the isotherm indicates a number of pores 4–8 nm in the samples (Figure 10B). With increasing etching time, the pore volume increases. A structure with abundant mesopores is likely to buffer the volume expansion and allow the penetration of electrolyte for complete contact with the active material, thus playing an important role in improving the electrochemical properties of α-Fe_2_O_3_@TiO_2_.

### Cyclic Voltammetry and Galvanostatic Cycling

**FIGURE 11:**
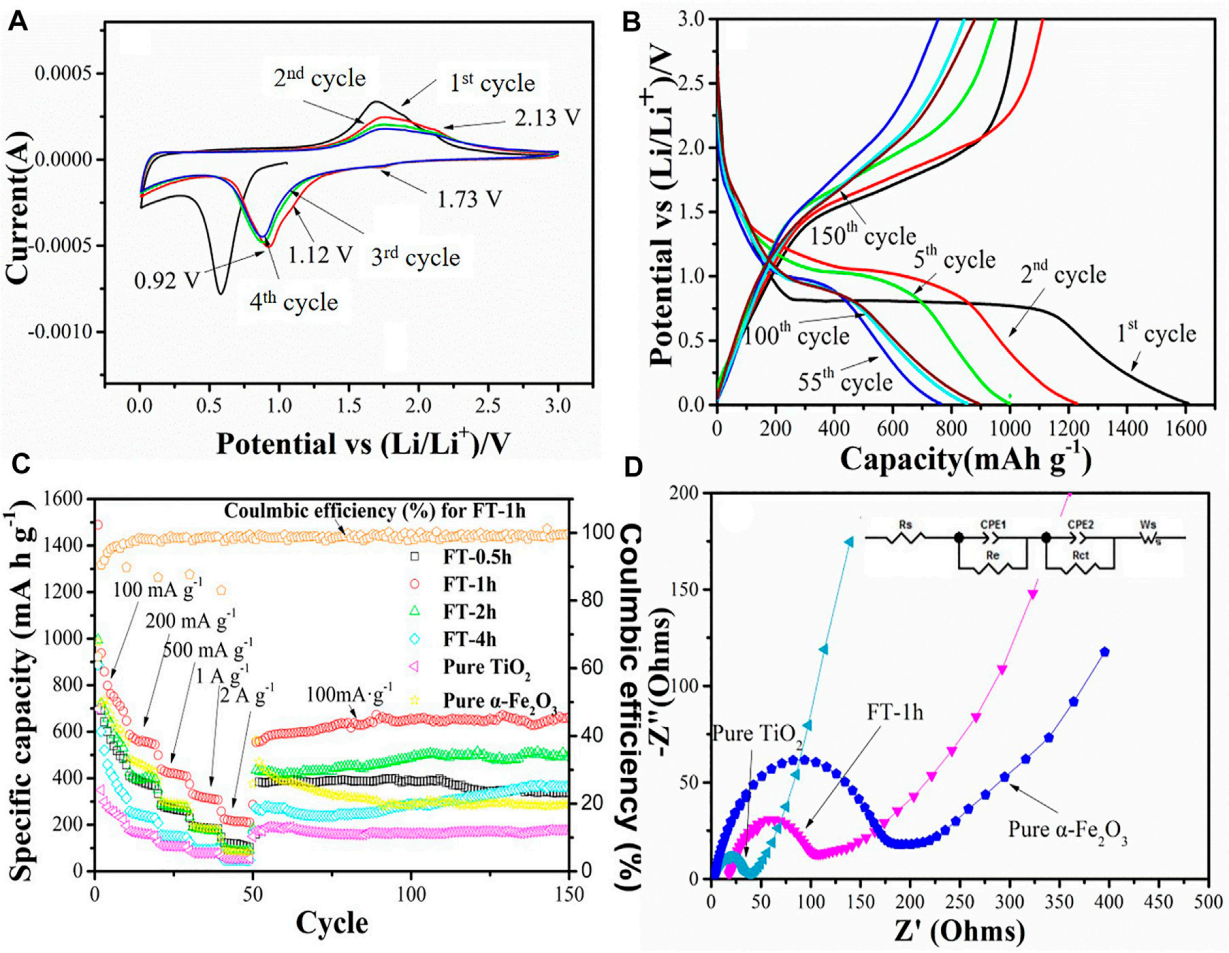
Electrochemical performance test diagram of different samples: **(A)** Cyclic voltammetry curve of the FT-1h sample in the voltage range of 0.01–3 V and a sweep rate of 0.1 mV s^−1^. **(B)** Charge–discharge test diagram of the FT-1h sample at a current density of 100 mA g^−1^. **(C)** Rate performance of oxalic acid-treated α-Fe_2_O_3_ coated with TiO_2_ and etched for different periods of time [FT-0.5h, FT-1h, FT-2h, FT-4h, and FT-12h (pure TiO_2_)], and the charge–discharge tests at a current density of 100 mA g^−1^ for each cell after the rate performance test. **(D)** EIS resistance test drawings of pure α-Fe_2_O_3_, pure TiO_2_, and FT-1h.

Electrochemical performances of α-Fe_2_O_3_@TiO_2_ (FT-1h) were investigated as anode materials for LIBs to demonstrate the effectiveness in improving lithium storage. Figure 11A shows the first four CV curves of the FT-1h sample between 0.01 and 3 V at a scan rate of 0.1 mV^−1^. It can be observed that the sample has a reduction peak and an oxidation peak at a potential of 1.73/2.13 V, which may be attributed to the de/insertion of lithium ions from TiO_2_, as shown in Eq. 2. There are two oxidation peaks at potentials of 0.92 and 1.12 V, which correspond to the lithium reaction of Fe_2_O_3_, respectively, as shown in Eq. 3 (Fu et al., 2015).

$${\text{Fe}}_{2}{\text{O}}_{3}+6{\text{Li}}^{+}6{\text{e}}^{-}\to 2{\text{Fe}}^{0}+3{\text{Li}}_{2}\text{O}\text{.}$$ (3)

In addition, the oxidation peak between 1.5 and 2.0 V may correspond to Fe^0^ being reoxidized to Fe_2_O_3_, as shown in Eq. 4 (Fu et al., 2015).

$${\text{Fe}}_{2}{\text{O}}_{3}+6\text{Li}↔2{\text{Fe}}^{0}+3{\text{Li}}_{2}\text{O}\text{.}$$ (4)

After the second charge–discharge cycle, the peak position slightly shifts, and the peak strength slightly weakens, which may be attributed to the polarization of the anode material and the formation of an SEI film during the charge–discharge process (Fu et al., 2015). It can be clearly observed that the curves of the fourth charge–discharge cycle and the third charge–discharge cycle coincide well, indicating that the FT-1h sample has good cycling stability.

Charge and discharge curves of the sample FT-1h with a voltage of 0.01–3 V are shown in Figure 11B. According to the previous analysis, a wide slope from 1.75 to 0.8 V is attributed to the Li^+^ intercalation into the crystal structure of α-Fe_2_O_3_ and the phase transformation from hexagonal to cubic Li_2_Fe_2_O_3_ (Qin et al., 2018). Last, an obvious plateau at 0.8 V is ascribed to the reduction from Fe^2+^ into Fe^0^, and the formation of amorphous Li_2_O and an SEI layer (Zhu et al., 2020). After the first charge and discharge cycle, the discharge/charge specific capacities of the FT-1h sample are 1,609.3 mA h g^−1^ and 1,021.6 mA h g^−1^, respectively. In the second process, a plateau at 1.0 V is observed, which agrees well with Eq. 3. Furthermore, the discharge specific capacity is reduced to 1,228.6 mA h g^−1^, and the Coulombic efficiency is 99.1%. The fifth circle is similar to the second, except the length of the plateau, which is shorter than the one in the first circle, and this is due to the irreversible capacity loss derived from the formation amorphous Li_2_O (Yang et al., 2019). The discharge specific capacity is 999.4 mA h g^−1^ after the fifth cycle. The 55th, 100th, and 150th curves express the similar circle curves, whose plateau is at 0.9 V, which is consistent with Eq. 3. After 150 charge–discharge cycles at a current density of 100 mA g^−1^, the reversible capacity remains at 893.7 mA h g^−1^, and the Coulombic efficiency is 98.4%.

Rate measurements of pure α-Fe_2_O_3_, oxalic acid-treated α-Fe_2_O_3_ coated with TiO_2_ and etched for different periods of time (FT-0.5h, FT-1h, FT-2h, FT-4h, and FT-12h (pure TiO_2_), and the charge–discharge tests at a current density of 100 mA g^−1^ for each cell after the rate performance test are shown in Figure 11C. The reversible capacities of FT-1h at current densities of 100 mA g^−1^, 200 mA g^−1^, 500 mA g^−1^, 1 A g^−1^, and 2 A g^−1^ reach 1,489.3, 586.8, 438.7, 332.7, and 224.5 mA h g^−1^, respectively. When the current density is returned to 100 mA g^−1^, the reversible capacity is still maintained at 555.5 mA h g^−1^. After 100 charge–discharge cycles at a current density of 100 mA g^−1^, the reversible capacity remains at 682.0 mA h g^−1^, and the Coulombic efficiency is 99.7%. During the long cycle, the Coulombic efficiency is maintained above 98%. The capacity decreases at the beginning and continuously increases during the latter long term cycle test. It is supposed to be the grain boundaries of iron metal and Li_2_O formed in the electrochemical process (Eq. 4), which contributes to the extra energy storage of the composite (Li S. et al., 2019). This phenomenon is common for most MO_*x*_ (M = Fe, Co, and Ni) attributed to the activation of the materials (Kaprans et al., 2018). Comparing the different etching times, it can be found that the battery performances of the etched samples are higher than that of pure α-Fe_2_O_3_ and TiO_2_, indicating that the different degrees of buffer chambers can relieve, to a certain extent, the volume expansion of α-Fe_2_O_3_ during the discharge/charge process. Furthermore, the SEM images of pure Fe_2_O_3_ and FT-1h after 100 charge–discharge cycles are shown in Supplementary Figure S3. Fe_2_O_3_ morphology gets damaged severely if TiO_2_ is not protected. On the contrary, sample FT-1h maintained the original morphology, which proved again that TiO_2_ can effectively inhibit the volume expansion. Among the etched samples, FT-1h possesses the best performance. Figure 11C shows that FT-1h still has good cycling performance after the large rate performance test, while the reversible capacities of pure α-Fe_2_O_3_ and pure TiO_2_ are low. The as-prepared α-Fe_2_O_3_@TiO_2_ in this work shows superior cyclability and capacity compared with others summarized in Table 1.

**TABLE 1:** List of the reported works on Fe_2_O_3_@TiO_2_ as anodes for lithium-ion batteries.

Material	Capacity/constant current density	Cycles	Ref.
Two-dimensional Fe_2_O_3_/TiO_2_ composite nanoplates	646 mA h g^−1^/1,000 mA g^−1^	1,000 cycles	Qu et al. (2020)
Clustered Fe_2_O_3_/TiO_2_ composite	792 mA h g^−1^/1,000 mA g^−1^	600 cycles	Chen et al. (2020)
TiO_2_/Fe_2_O_3_ nanotubular composite	571 mA h g^−1^/100 mA g^−1^	150 cycles	Li et al. (2019a)
Fe_2_O_3_ and TiO_2_ nanograins anchored on rGO layers	790 mA h g^−1^/100 mA g^−1^	150 cycles	Kaprans et al. (2018)
α-Fe_2_O_3_/TiO_2_ composite	638 mA h g^−1^/33.5 mA g^−1^	30 cycles	Qi et al. (2018)
Fe_2_O_3_@TiO_2_ core–shell nanospheres	497 mA h g^−1^/100 mA g^−1^	100 cycles	Qin et al. (2018)
α-Fe_2_O_3_@TiO_2_ core–shell structures with tunable buffer chambers	893.7 mA h g^−1^/100 mA g^−1^	150 cycles	This work

EIS resistance tests are conducted for pure α-Fe_2_O_3_, pure TiO_2_, and FT-1h, and the Nyquist plots of FT-1h, pure α-Fe_2_O_3_, and pure TiO_2_ are shown in Figure 11D. All of them exhibit a similar profile. There is a depressed semicircle in the high-frequency region and an inclined line in the low-frequency region. The diameter of the semicircle in the high-frequency region of each cell is related to the tenable resistance of the electrolyte (*R*_*e*_), SEI layer (*R*_*s*_), and charge transfer resistance (*R*_*ct*_) (Wang et al., 2020). *R*_*ct*_ is associated with the transfer of electrons and Li^+^. The inclined line in the low-frequency region represents the Warburg impedance (*Z*_*w*_), which is derived from lithium-ion diffusion in electrode materials. A semicircle with a larger diameter in the high-frequency region represents a larger *R*_*ct*_ (Yang et al., 2019). From Figure 11D, it can be found that the impedance of α-Fe_2_O_3_ alone is relatively large, and the impedance decreases after coating with TiO_2_, indicating that the electrical conductivity of the electrode is improved by forming a composite material. In addition, the higher slope of FT-1h compared with pure α-Fe_2_O_3_ in the low-frequency region is evidence of a stronger interaction between Li ions and FT-1h.

## Conclusion

In summary, the volume expansion rates of α-Fe_2_O_3_ and TiO_2_ during Li-ion insertion were estimated through DFT calculations. The expansion rate of α-Fe_2_O_3_ with Li-ion insertion is clearly higher than that of TiO_2_, indicating that TiO_2_ could effectively alleviate crystal expansion when used in Li-ion batteries. Hence, to buffer the bulk expansion of α-Fe_2_O_3_ that occurs during the discharge/charge process, cubic α-Fe_2_O_3_ was coated with a TiO_2_ layer (α-Fe_2_O_3_@TiO_2_) using the hydrothermal method, and then a buffer chamber was deliberately designed by immersing in hydrochloric acid for etching. It is found that α-Fe_2_O_3_@TiO_2_ with a buffer chamber structure could effectively relieve the volume expansion. Due to these structural advantages, the optimized FT-1h sample exhibits high reversible capacities of 1,489.3, 586.8, 438.7, 332.7, and 224.5 mA h g^−1^ at 100 mA g^−1^, 200 mA g^−1^, 500 mA g^−1^, 1 A g^−1^, and 2 A g^−1^, respectively. When the current density is returned to 100 mA g^−1^, the reversible capacity remains unchanged at 555.5 mA h g^−1^. Thanks to the buffer chambers, FT-1h demonstrates good cycling performance. After 150 charge–discharge cycles at a current density of 100 mA g^−1^, the reversible capacity is 893.7 mA h g^−1^, the Coulombic efficiency is 98.4%, and the morphology is in a good condition. Furthermore, the results obtained in this study provide new insights into the synthesis of metal oxide-based LIB anode materials with well-designed structures.

## Data Availability

The original contributions presented in the study are included in the article/Supplementary Material, and further inquiries can be directed to the corresponding authors.

## Supplementary Material

Supplementary Figure S1 Unit cells of α-Fe_2_O_3_ **(A)** and TiO_2_ **(B)**.

Supplementary Figure S2 XRD of α-Fe_2_O_3_.

Supplementary Figure S3 SEM images of pure Fe_2_O_3_ **(A,B)** and FT-1h **(C,D)** after 100 charge–discharge cycles.

Supplementary Table S1 Data compared with other theoretical and experimental data for α-Fe_2_O_3_ and TiO_2._

Supplementary Table S2 Volume (V) and expansion rate (E_r_) of the one, two, three, four, and five and one, second, and three Li ions inserted in the cell at different sites of α-Fe_2_O_3_ and TiO_2_.,

Supplementary Table S3 Electronic population of Fe-O and Ti-O bonds for α-Fe_2_O_3_ and TiO_2_ with and without Li inserted at symmetrical sites.

Supplementary Table S4 Average core–shell size of oxalic acid-treated α-Fe_2_O_3_ coated with TiO_2_ and etched for different periods of time (FT-0.5h (A-C), FT-2h (D-F), FT-4h (G-I), and FT-12h (J-L) (pure TiO_2_).
