# Supplementary material for: Robust α-Fe2O3@TiO2 Core–Shell Structures With Tunable Buffer Chambers for High-Performance Lithium Storage
Source: Front Chem. 2022 Apr 7;10:866369. doi: 10.3389/fchem.2022.866369 (PMC9021487; doi:10.3389/fchem.2022.866369)
Supplement: Supplementary file 1 [file DataSheet1.DOC]

**Table S1** The present data compared with other theoretical and experimental data for α-Fe2O3 and TiO2.

|  | a (Å) | c (Å) |
| --- | --- | --- |
| α-Fe_2_O_3_ |  |  |
| present | 4.75 | 13.44 |
| Theo. | 5.14 | 13.73 |
| Exp. | 5.04 | 13.747 |
| TiO_2_ |  |  |
| present | 3.80 | 9.69 |
| Theo. | 3.78 | 9.49 |
| Exp. | 3.79 | 9.51 |

**Table S2** The Volume (V) and expansion rate (E_r_) of the 1, 2, 3, 4, 5 and 1, 2, 3 Li ions inserted in the cell at different sites of α-Fe_2_O_3_ and TiO_2_.

|  | V | E_r_ |
| --- | --- | --- |
| α-Fe_2_O_3_+Li | 267.13 | 1.74% |
| α-Fe_2_O_3_+2Li(1) | 271.55 | 3.42% |
| α-Fe_2_O_3_+2Li (2) | 271.60 | 3.44% |
| α-Fe_2_O_3_+2Li (3) | 271.41 | 3.37% |
| α-Fe_2_O_3_+3Li (1) | 275.94 | 5.09% |
| α-Fe_2_O_3_+3Li (2) | 275.72 | 5.01% |
| α-Fe_2_O_3_+3Li (3) | 275.74 | 5.02% |
| α-Fe_2_O_3_+3Li (4) | 275.93 | 5.09% |
| α-Fe_2_O_3_+3Li (5) | 275.75 | 5.02% |
| α-Fe_2_O_3_+3Li (6) | 276.08 | 5.15% |
| α-Fe_2_O_3_+4Li (1) | 280.00 | 6.64% |
| α-Fe_2_O_3_+4Li (2) | 280.12 | 6.69% |
| α-Fe_2_O_3_+4Li (3) | 279.94 | 6.62% |
| α-Fe_2_O_3_+4Li (4) | 280.11 | 6.68% |
| α-Fe_2_O_3_+4Li (5) | 279.98 | 6.63% |
| α-Fe_2_O_3_+4Li (6) | 276.08 | 5.15% |
| α-Fe_2_O_3_+5Li (1) | 284.09 | 8.20% |
| α-Fe_2_O_3_+5Li (2) | 284.09 | 8.20% |
| α-Fe_2_O_3_+6Li | 287.89 | 9.64% |
| TiO_2_+Li(1) | 142.34 | 1.49% |
| TiO_2_+Li(2) | 143.07 | 2.01% |
| TiO_2_+2Li(1) | 143.27 | 2.15% |
| TiO_2_+2Li (2) | 141.84 | 1.14% |
| TiO_2_+2Li (3) | 143.32 | 2.19% |
| TiO+2Li (4) | 143.02 | 1.98% |
| TiO_2_+3Li(1) | 141.46 | 0.86% |
| TiO_2_+3Li (2) | 142.12 | 1.34% |
| TiO_2_+3Li (3) | 142.12 | 1.34% |
| TiO_2_+4Li(4) | 141.85 | 1.15% |


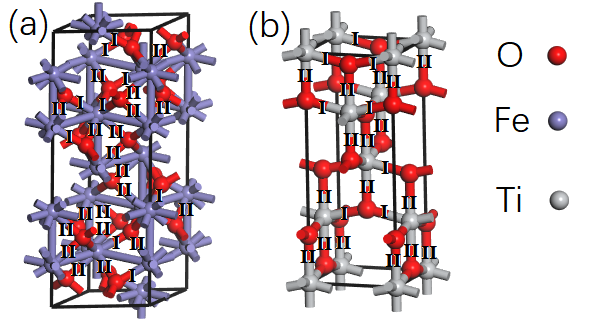


**Figure S1 |** The unit cells of α-Fe_2_O_3_ (a) and TiO_2_(b).

**Table S3** Electronic population of Fe-O and Ti-O bonds for α-Fe_2_O_3_ and TiO_2_ with Li inserted in symmetrical sites and not.

| α-Fe_2_O_3_ | | | α-Fe_2_O_3_+6Li | | | TiO_2_ | | | TiO_2_+4Li | | |
| --- | --- | --- | --- | --- | --- | --- | --- | --- | --- | --- | --- |
| Bond | Electronic population | Bond length | Bond | Electronic population | Bond length | Bond | Electronic population | Bond length | Bond | Electronic population | Bond length |
| Fe-O I | 0.31 | 1.883 | Fe-O I | 0.38 | 1.991 | Ti-O I | 0.71 | 1.947 | Ti-O I | 1.07 | 2.039 |
| Fe-O II | 0.26 | 1.979 | Fe-O II | 0.29 | 1.987 | Ti-O II | 0.29 | 2.005 | Ti-O II | 0.32 | 2.117 |

**Table S4** The average core-shell size of oxalic acid-treated α-Fe_2_O_3_ coated with TiO_2_ and etched for different periods of time (FT-0.5h (A-C), FT-2h (D-F), FT-4h (G-I), FT-12h (J-L) (pure TiO_2_).

| Samples | Shell | Core | |
| --- | --- | --- | --- |
|  | thickness | Diagonal1 | Diagonal2 |
| FT-0.5h | 58 | 560 | 568 |
| FT-1h | 53 | 323 | 534 |
| FT-2h | 53 | 248 | 519 |
| FT-4h | 53 | 100 | 419 |
| FT-12h | 45 | - | - |


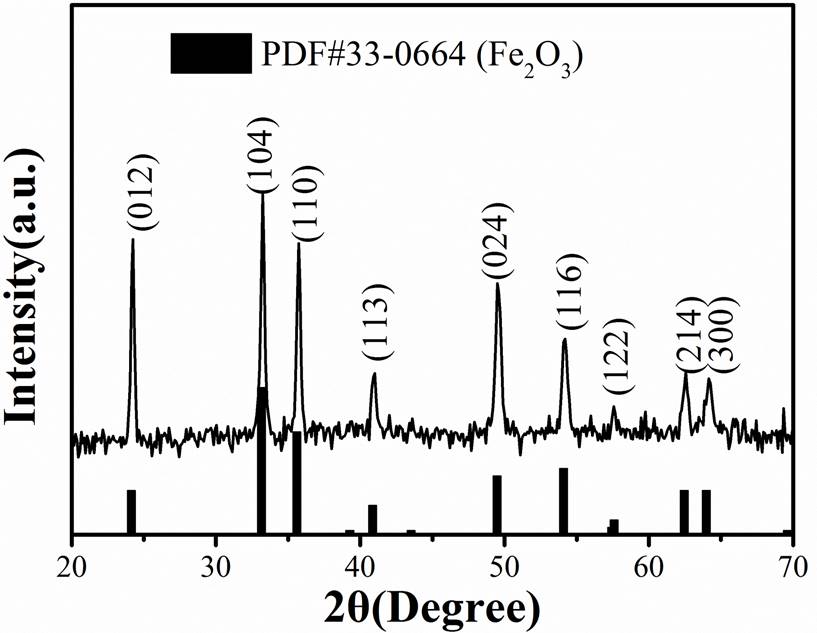


**Figure S2 |** XRD of α-Fe_2_O_3_


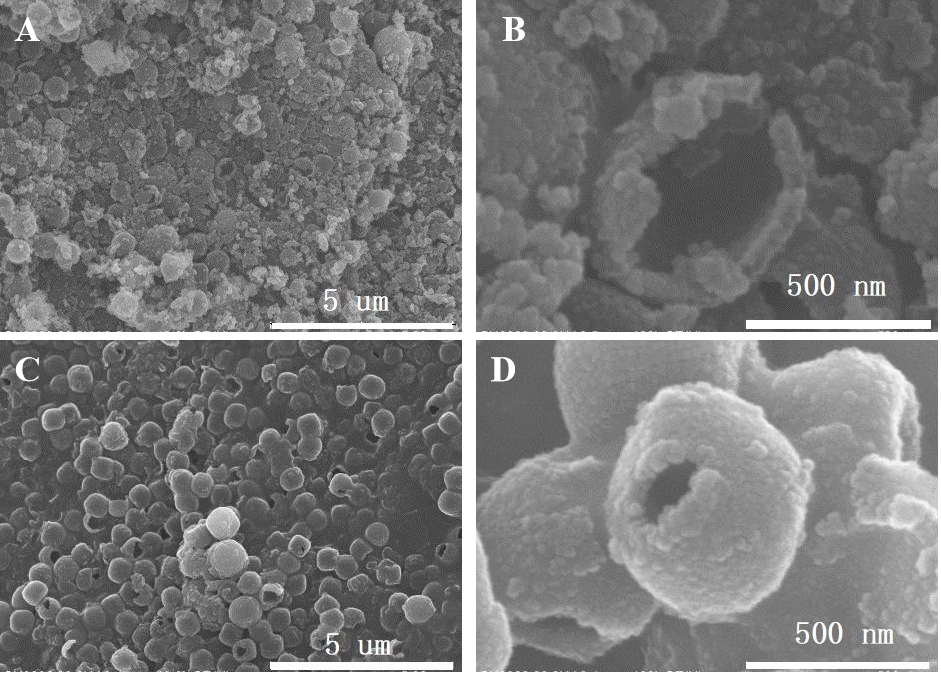


**Figure S3 |** SEM images of pure Fe_2_O_3_ **(A, B)** and FT-1h **(C, D)** after 100 charge-discharge cycles.

**Reference**

[1] Glen R. Jenness, Jennifer Seiter, Manoj K. Shukla, DFT investigation on the adsorption of munition compounds on α-Fe2O3: similarity and differences with α-Al2O3, Physical Chemistry Chemical Physics 20 (2018) 18850.

[2] Larry W. Finger, Robert M. Hazen, Crystal structure and isothermal compression of Fe2O3, Cr2O3, and V2O3 to 50 kbars, Journal of Applied Physics 51 (1980) 5362.

[3] Hua-Xi Zhang, Yong Fu Zhu, Ming Zhao, Interface charge transfer and enhanced visible light response of graphene/anatase TiO 2 (110) systems with and without oxygen vacancy: A DFT+U calculation, Applied Surface Science 420 (2017) 105.

[4] T. Arlt, M. Bermejo, M. A. Blanco, L. Gerward, J. Z. Jiang, J. Staun Olsen, J. M. Recio, High-pressure polymorphs of anataseTiO2, Physical Review B 61 (2000) 14414.
